# Improvement for enhanced xylanase production by *Cellulosimicrobium cellulans* CKMX1 using central composite design of response surface methodology

**DOI:** 10.1007/s13205-015-0309-2

**Published:** 2015-06-03

**Authors:** Abhishek Walia, Preeti Mehta, Shiwani Guleria, Chand Karan Shirkot

**Affiliations:** Department of Microbiology, DAV University, Jalandhar, 144001 Punjab India; Centre for Advance Bioenergy Research, Research & Development Centre, Indian Oil Corporation Limited, Sector-13, Faridabad, 121 007 India; Department of Basic Sciences, Dr. Y. S. Parmar University of Horticulture and Forestry, Nauni, Solan, 173230 Himachal Pradesh India

**Keywords:** Xylanase, *Cellulosimicrobium cellulans* CKMX1, Optimization, Response surface methodology, Central composite design, Biobleaching

## Abstract

The effects of yeast extract (X_1_), NH_4_NO_3_ (X_2_), peptone (X_3_), urea (X_4_), CMC (X_5_), Tween 20 (X_6_), MgSO_4_ (X_7_), and CaCO_3_ (X_8_) on production of xylanase from *Cellulosimicrobium cellulans* CKMX1 were optimized by statistical analysis using response surface methodology (RSM). The RSM was used to optimize xylanase production by implementing the Central composite design. Statistical analysis of the results showed that the linear, interaction and quadric terms of these variables had significant effects. However, only the linear effect of X_4_, X_5_, interaction effect of X_1_X_7_, X_1_X_8_, X_2_X_3_, X_2_X_8_, X_3_X_6_, X_3_X_8_, X_4_X_6_, X_4_X_7_, X_5_X_7_, X_5_X_8_ and quadratic effect of X_3_^2^, X_5_^2^ and X_7_^2^ found to be insignificant terms in the quadratic model and had no response at significant level. The minimum and maximum xylanase production obtained was 331.50 U/g DBP and 1027.65 U/g DBP, respectively. The highest xylanase activity was obtained from Run No. 30, which consisted of yeast extract (X_1_), 1.00 g (%); NH_4_NO_3_ (X_2_), 0.20 g (%); peptone (X_3_), 1.00 g (%); urea (X_4_), 10 mg (%); CMC (X_5_), 1.00 g (%); Tween 20 (X_6_), 0.02 mL (%); CaCO_3_ (X_7_), 0.50 g (%) and MgSO_4_ (X_8_), 9.0 g (%). The optimization resulted in 3.1**-**fold increase of xylanase production, compared with the lowest xylanase production of 331.50 U/g DBP after 72 h of incubation in stationary flask experiment. Application of cellulase-free xylanase in pulp biobleaching from *C. cellulans* CKMX1 under C–E_P_–D sequence has been shown to bring about a 12.5 % reduction of chlorine, decrease of 0.8 kappa points (40 %), and gain in brightness was 1.42 % ISO points in 0.5 % enzyme treated pulp as compared to control.

## Introduction

Xylan is the major hemicellulosic constituent of hard and soft wood and is the next most abundant renewable polysaccharide after cellulose. This complex heteropolysaccharide consists of a main chain of 1,4-β-d-xylose monomers and short chain branches consisting of *O*-acetyl, α-l-arabinofuranosyl and α-d-glucuronyl residues. Xylanases and associated debranching enzymes produced by a variety of microorganisms including bacteria, yeast and filamentous fungi, bring about the hydrolysis of hemicelluloses (Maheshwari et al. 2000). Xylanolytic enzymes are receiving increasing attention because of their potential application in pulp bleaching (Goluguri et al. 2012; Singh et al. 2013) and bioconversion of lignocelluloses into feedstocks and fuels (Kim et al. 2000). The xylan degrading system includes endo-1,4-xylanases (1,4-β-xylan xylanohydrolase; EC 3.2.1.8), which release long and short xylo-oligosaccharides, and other xylanases that attack only longer chains, and β-d-xylosidase (1,4-β-xylan xylohydrolase; EC 3.2.1.37), which remove d-xylose residues from short xylo-oligosaccharides (Gomez et al. 2008; Saha 2003).

Cellulase-free xylanases are important in the paper and pulp industry as alternatives to the use of toxic chlorinated compounds (Li et al. 2010; Woldesenbet et al. 2012; Walia et al. 2014). For the last two decades the bleaching of pulp has become an issue of great concern, primarily because of the environmental hazards caused by the release of the adsorbable organic halogens and due to increasing public awareness thereof (Goluguri et al. 2012).

The commercial application of xylanase in various industrial processes has had limited success due to various factors. These include inaccessibility of substrate to xylanase enzymes because of physical limitations, the limited hydrolysis of xylans due to their branched nature, thermal instability, narrow pH range, end product inhibition and cost of enzyme production. The last two problems can be overcome to some extent by the use of cheap substrates and by employing the process of solid-state fermentation (SSF).

*Cellulosimicrobium cellulans* CKMX1 grows well on apple pomace in solid-state culture conditions and produces a high level of xylanase (Walia et al. 2013). The optimal culture medium for this strain for SSF has not yet been developed and designing such a medium would improve significantly the yield and quality of xylanase. There are two ways by which the problem of fermentation parameters may be addressed: classical and statistical. The classical method is based on the “one-factor-at-a-time” method in which one independent variable is studied while maintaining all the other factors at a fixed level (Li et al. 2007a; Khucharoenphaisan et al. 2008). This method may lead to unreliable results, inaccurate conclusions and requires a considerable amount of work and time. Moreover, it does not guarantee the determination of optimal conditions and is unable to detect the frequent interactions occurring between two or more factors. An alternate strategy is a statistical approach, e.g. factorial experimental design and response surface methodology (RSM) (Coman and Bahrim 2011), involving a minimum number of experiments for a large number of factors to determine and simultaneously solve multivariate equations, by which improvement in enzyme production has been demonstrated successfully (Katapodis et al. 2007; Ellouze et al. 2008; Khucharoenphaisan et al. 2008).

Cultural conditions and process parameters i.e. type of medium, particle size of carbon source, incubation period, temperature, initial pH, inoculum size and nutritional parameters were optimized using classical approach i.e. one-factor-at-a-time approach in previous work and xylanase activity was increased to 570.0 U/g DBP. CMCase, avicelase, FPase and β-glucosidase activities were not detected, highlighting the novelty of the xylanase enzyme produced by CKMX1. Therefore, keeping in view the importance of statistical approach, an attempt has been made to improve the production of xylanase using central composite design (CCD) following RSM with eight independent variables as additional sources of carbon and nitrogen with apple pomace as cheap agricultural waste by *C. cellulans* CKMX1 in SSF.

## Materials and methods

### Microorganism

#### Strain

 A bacterial culture isolated originally from mushroom compost and was characterized by metabolic fingerprinting, whole-cell fatty acids methyl ester analysis and 16S rDNA and found to be *C. cellulans* CKMX1. The 16S rDNA sequence of the strain has been deposited in the GenBank database under accession number JN135476.

#### Culture and growth medium

The bacterial culture was grown and maintained in basal salt medium (BSM, pH 8.0) containing 0.5 % xylan with the following composition (g/L): Na_2_HPO_4_, 6.0; KH_2_PO_4_, 3.0; NaCl, 0.5; NH_4_Cl, 1.0, 1 M MgSO_4_ (2 mL) and 1 M CaCl_2_ (0.1 mL). The bacterial culture was maintained in 30 % glycerol at −20 °C.

#### Apple pomace as substrate

Apple pomace was procured from the processing unit of the Horticultural Produce Marketing and Processing Corporation (HPMC; Parwanoo, Himachal Pradesh, India). The ovendried material (60 °C for 48 h) was ground in an electric grinder and packed in air-tight containers for subsequent studies.

#### Enzyme production and extraction

Solid-state fermentation was carried out in Erlenmeyer flasks (250 mL) containing 10 g substrate (apple pomace) and 20 mL mineral salt solution (BSM) at pH 8.0 were autoclaved at 15 psi pressure for 20 min, cooled and inoculated with 2 mL bacterial suspension (OD 1.0 at 540 nm).After mixing, the flasks were incubated at 35 °C for 3 days. At the desired intervals, the flasks were taken out, and the contents were extracted with 50 mL sterilized buffer (0.2 M, pH 8.0, Tris HCl). The flasks were kept in shaker for half an hour to ensure thorough mixing of apple pomace with the buffer. The flask contents were centrifuged at 8000 rpm for 20 min at 4 °C. The culture supernatant was used as crude enzyme preparation (prior to centrifugation, samples were withdrawn for determining viable number of cells by the standard viable plate count technique).

#### Enzyme assay

Xylanase activity was assayed using 1 % oat spelt xylan (Sigma, St, Louis, MO) in 0.2 M Tris–HCl buffer (pH 8.0) according to the calorimetric method of Miller (1959). The release of reducing sugars was determined using the 3,5-dinitrosalicylic acid (DNSA) method with a xylose standard curve. The reaction mixture contained 0.5 mL 1 % d-xylanin Tris–HCl buffer (0.2 M, pH 8.0) and 0.5 mL diluted enzyme. It was incubated at 50 °C for 5 min in a water bath with occasional shaking. After incubation, 3 mL DNSA reagent was added into the test tubes, which also stopped the enzymatic reaction. The tubes were immersed in boiling water bath and removed after 15 min when colour development was completed. Tubes were cooled to room temperature. The contents were transferred to a 25 mL volumetric flask and final volume made up with distilled water. Optical density was read at 540 nm in a Spectronic-20.

One unit (IU) of enzyme activity was defined as the amount of enzyme required to liberate 1 μmol reducing sugars per minute under given assay conditions. Xylanase activity is expressed as U/g dry bacterial pomace (DBP).

### Response surface methodology

Using the ‘one variable at a time’ approach, the effect of media types, particle size of carbon source, incubation temperature, pH, incubation time, moisture level, inoculum size, yeast extract, NH_4_NO_3_, peptone, urea, carboxymethyl cellulose (CMC), Tween 20, CaCO_3_ and MgSO_4_ were studied previously. Based on these experiments, eight independent variables were chosen for further optimization by RSM using CCD experiments. The dependent variable selected for this study were the enzyme activity, reducing sugar, viable count, pH, extracellular protein, specific activity and the independent variables chosen were yeast extract (X_1_), NH_4_NO_3_ (X_2_), peptone (X_3_), urea (X_4_), CMC (X_5_), Tween 20 (X_6_), CaCO_3_ (X_7_) and MgSO_4_ (X_8_). Each variable was studied at three levels (−1, 0, +1). The range and the levels of these variables are given in Table 2. The experimental design included 51 flasks with three replicates at their central coded values (Chadha et al. 2004). The mathematical relationship of response (enzyme production) and variable X_1_, X_2_, X_3_, X_4_, X_5_, X_6_, X_7_ and X_8_ was approximated by a quadratic model equation. The response value in each trial is the average of triplicate experiments.

### Central composite design

RSM was used to optimize the fermentation parameters for enhancing xylanase production. RSM has four steps: procedures to move into the optimum region, behaviour of the response in the optimum region, estimation of the optimal condition and verification (Tanyildizi et al. 2005). A CCD (Box and Wilson 1951; Coman and Bahrim 2011) was employed in this study. According to the CCD, the total number of experimental combinations is 2^*k*^ + 2*k* + *n*_0_, where *k* is the number of independent variables and *n*_0_ is the number of repetitions of the experiments at the centre point. For statistical calculation, the experimental variables *X*_*i*_ have been coded as *x*_*i*_ according to the following transformation equation

$$x_{i} = \frac{{X_{i} - X_{0} }}{\delta X}$$

where *x*_*i*_ is the dimensionless coded value of the variable *X*_*i*_, *X*_0_ is the value of *X*_*i*_ at the centre point, and *δX* is the step change.

In this study, the CCD with eight factors and three levels, including three replicates at the centre point, was used for fitting a second order response surface. Table 2 gives the factors and their values, respectively. This methodology allows the modelling of a second order equation that describes the process. Xylanase production was analysed by multiple regression through the least squares method to fit the following equation:

$$Y = A_{0} + \sum AiXi + \sum AiiXi + \sum AijXiXj$$

where *Y* is the predicted response variable; *A*_0_, *Ai*, *Aii*, *Aij* are constant regression coefficients of the model, and *Xi*, *Xj* (*i* = 1, 3; *j* = 1, 3, *i* ≠ *j*) represent the independent variables (medium composition) in the form of coded values. The accuracy and general ability of the above polynomial model could be evaluated by the coefficient of determination *R*^2^. Each experimental design was carried out in triplicate, and the mean values were given.

### Statistical analysis

The statistical software package Design-Expert 8.0.4 (StatEase, Minneapolis, MN) was used for regression analysis of experimental data to obtain working parameters and to generate response surface graphs. ANOVA was used to estimate statistical parameters.

## Results

### Regression model of response

In this method, prior knowledge obtained from a previous experiment i.e. one-factor-at-a-time approach (understanding of the cultivation condition variables under investigation) was necessary for achieving a more realistic model (Data not shown). Table 1 shows the maximum and minimum levels of variables chosen for trials (Run) in the CCD. For RSM based on the CCD, used for the optimization of independent variables for the xylanase production, 51 experimental runs with different combinations of eight factors were carried out. The variables used for the factorial analysis were yeast extract (X_1_), ammonium nitrate (X_2_), peptone (X_3_), urea nitrogen (X_4_), CMC (X_5_), Tween 20 (X_6_), CaCO_3_ (X_7_) and MgSO_4_ (X_8_). The range and the levels of these variables are given in Table 2. The experimental responses for the 51 runs are presented in Table 2, which shows considerable variation in the amount of xylanase production depending on the eight independent variables in the medium.

**Table 1:** Coded values of independent variables at different levels used in central composite design

Independent variables	Symbol	Levels		
		−1	0	+1
Yeast extract g (%)	X_1_	0.20	0.60	1.00
Ammonium nitrate g (%)	X_2_	0.20	0.60	1.00
Peptone g (%)	X_3_	0.20	0.60	1.00
Urea nitrogen mg (%)	X_4_	10.0	30.0	50.0
CMC g (%)	X_5_	1.00	3.00	5.00
Tween 20 mL (%)	X_6_	0.20	0.60	1.00
CaCO_3_ g (%)	X_7_	0.50	1.00	1.50
MgSO_4_ g (%)	X_8_	1.00	5.00	9.00

**Table 2:** Actual and predicted values of xylanase recorded in experimental setup of response surface methodology

Std	Run	Yeast extract	NH_4_NO_3_	Peptone	Urea	CMC	Tween 20	CaCO_3_	MgSO_4_	Xylanase activity (U/g DBP) actual	Xylanase activity (U/g DBP) predicted
45	1	0	0	0	0	0	0	0	−1	618.79	644.62
47	2	0	0	0	0	0	0	0	0	647.65	658.50
18	3	−1	−1	+1	+1	+1	−1	−1	+1	386.75	390.50
36	4	0	0	+1	0	0	0	0	0	718.25	710.15
43	5	0	0	0	0	0	0	−1	0	408.85	434.68
42	6	0	0	0	0	0	+1	0	0	685.09	710.92
2	7	−1	+1	+1	−1	+1	+1	+1	−1	861.90	852.60
38	8	0	0	0	+1	0	0	0	0	729.30	755.10
34	9	0	+1	0	0	0	0	0	0	568.75	562.84
1	10	+1	+1	+1	−1	−1	+1	+1	+1	663.00	673.92
31	11	−1	0	0	0	0	0	0	0	607.75	633.58
49	12	0	0	0	0	0	0	0	0	625.49	637.07
29	13	−1	+1	−1	+1	−1	−1	−1	+1	475.15	471.72
21	14	+1	+1	−1	+1	−1	−1	+1	−1	497.25	505.61
7	15	−1	+1	+1	+1	+1	+1	−1	−1	596.70	614.35
27	16	−1	−1	−1	−1	+1	−1	+1	+1	817.69	776.39
24	17	+1	−1	−1	−1	+1	+1	+1	+1	397.80	407.03
20	18	−1	−1	+1	+1	−1	+1	+1	+1	419.89	433.30
40	19	0	0	0	0	+1	0	0	0	593.05	590.05
50	20	0	0	0	0	0	0	0	0	641.45	637.07
30	21	−1	−1	−1	−1	−1	−1	−1	−1	386.75	380.66
33	22	0	−1	0	0	0	−1	0	0	873.49	870.07
3	23	+1	+1	−1	−1	+1	+1	−1	−1	563.55	545.83
28	24	+1	−1	+1	+1	+1	−1	+1	−1	530.40	529.53
51	25	0	0	0	0	0	−1	0	0	627.85	637.07
5	26	−1	−1	+1	+1	+1	+1	−1	−1	839.79	813.65
44	27	0	0	0	0	0	0	+1	0	773.49	799.32
26	28	−1	+1	−1	+1	+1	+1	+1	+1	994.50	976.78
9	29	+1	+1	−1	+1	−1	+1	−1	+1	386.75	401.03
14	30	+1	−1	+1	−1	−1	−1	−1	+1	1027.65	1041.93
11	31	+1	+1	+1	−1	+1	−1	−1	+1	685.09	666.49
17	32	−1	−1	−1	−1	−1	+1	+1	−1	850.85	861.77
41	33	0	0	0	0	0	−1	0	0	331.50	357.33
32	34	+1	0	0	0	0	0	0	0	508.29	534.12
39	35	0	0	0	0	−1	0	0	0	605.05	588.72
6	36	−1	+1	+1	+1	−1	+1	−1	−1	607.75	464.10
25	37	+1	−1	+1	−1	−1	+1	+1	+1	884.00	693.79
4	38	+1	−1	−1	+1	+1	−1	+1	−1	386.75	365.66
46	39	0	0	0	0	0	0	0	+1	530.40	556.23
35	40	0	0	−1	0	0	0	0	0	579.35	595.71
22	41	−1	−1	+1	−1	+1	−1	−1	−1	497.25	493.00
8	42	+1	+1	+1	+1	+1	−1	+1	−1	651.95	651.08
23	43	+1	+1	+1	−1	−1	+1	−1	−1	563.55	589.62
10	44	+1	−1	+1	+1	−1	−1	+1	−1	386.75	392.61
48	45	0	0	+1	0	0	0	0	0	617.65	637.07
19	46	−1	+1	0	−1	+1	−1	+1	+1	497.25	516.59
13	47	+1	−1	−1	−1	−1	+1	−1	−1	607.75	595.09
15	48	−1	+1	−1	−1	+1	+1	+1	−1	718.25	712.32
37	49	0	0	−1	−1	0	0	0	0	718.25	744.08
12	50	−1	+1	0	−1	−1	−1	+1	+1	552.49	570.14
16	51	+1	−1	+1	+1	+1	+1	−1	+1	386.75	395.09

As shown in Table 2, the minimum and maximum xylanase production obtained was 331.50 U/g DBP and 1027.65 U/g DBP, respectively. The highest xylanase activity was obtained from Run No. 30, which consisted of Yeast extract, 1.00 g (%); NH_4_NO_3_, 0.20 g (%); Peptone, 1.00 g (%); Urea nitrogen, 10 mg (%); CMC, 1.00 g (%); Tween 20, 0.02 mL (%); CaCO_3_, 0.50 g (%) and MgSO_4_, 9.0 g (%); while the lowest activity was obtained in Run No. 33, which consisted of Yeast extract, 0.60 g (%); NH_4_NO_3_, 0.60 g (%); Peptone, 0.60 g (%); Urea nitrogen, 30 mg (%); CMC, 3.0 g (%); Tween 20, 0.02 mL (%); CaCO_3_, 1.00 g (%) and MgSO_4_, 5.00 g (%). Other responses observed for maximum xylanase production (Run No. 30) are reducing sugars, 770.74 mg/mL/g; protein, 80.68 mg/mL; specific activity, 12.73 U/mg protein; viable count, 236.0 × 10^5^ cfu/mL and final pH 5.95, while for minimum xylanase production the other responses are reducing sugars, 248.63 mg/mL/g; protein, 65.69 mg/mL; specific activity, 5.05 U/mg protein; viable count, 144 × 10^5^ cfu/mL and final pH 5.96. Treatment runs were repeated three times for estimation of error. This result suggests that the data were deviated and the flask experiments were accurate. In general, the highest xylanase activity was obtained in medium supplemented with high level of yeast extract, peptone and MgSO_4_.

By applying multiple regression analysis on the experimental data, the following quadratic model was generated for the response of xylanase activity. The significant model terms were evaluated by ANOVA in the optimization study (Table 3) (*P* < 0.05) and were identified as X_1_, X_2_, X_3_, X_6_, X_7_, X_8_, X_1_X_2_, X_1_X_3_, X_1_X_4_, X_1_X_5_, X_1_X_6_, X_2_X_4_, X_2_X_5_, X_2_X_6_, X_2_X_7_, X_3_X_4_, X_3_X_5_, X_3_X_7_, X_4_X_5_, X_4_X_8_, X_5_X_6_, X_6_X_7_, X_6_X_8_, X_7_X_8_, X_1_^2^, X_2_^2^, X_4_^2^, X_6_^2^, X_8_^2^. From the analysis, only the linear effect of X_4_, X_5_ and interaction effect of X_1_X_7_, X_1_X_8_, X_2_X_3_, X_2_X_8_, X_3_X_6_, X_3_X_8_, X_4_X_6_, X_4_X_7_, X_5_X_7_, X_5_X_8_ and quadratic effect of X_3_^2^, X_5_^2^, X_7_^2^ found to be insignificant terms in the quadratic model. The model was reconstructed by removing the insignificant terms and is present in Eq. 1 as coded factors:

**Table 3:** ANOVA for response surface quadratic model (xylanase activity)

Source	Sum of squares	df	Mean square	F-value	*P* value (Prob > F)
Model	1.300E+006	44	29,546.55	47.03	<0.0001 significant
X_1_-Yeast Extract	4946.15	1	4946.15	7.87	0.0309^a^
X_2_-Amm. Nitrate	72,674.88	1	72,674.88	115.67	<0.0001^a^
X_3_-Peptone	7362.14	1	7362.14	11.72	0.0141^a^
X_4_-Urea Nitrogen	61.05	1	61.05	0.097	0.7658
X_5_-CMC	1784.86	1	1784.86	2.84	0.1429
X_6_-Tween 20	62,512.94	1	62,512.94	99.50	<0.0001^a^
X_7_ G-CaCO3	66,481.16	1	66,481.16	105.82	<0.0001^a^
X_8_-MgSO4	3906.40	1	3906.40	6.22	0.0469^a^
X_1_X_2_	16,266.75	1	16,266.75	25.89	0.0022^a^
X_1_X_3_	46,580.68	1	46,580.68	74.14	0.0001^a^
X_1_X_4_	26,501.28	1	26,501.28	42.18	0.0006^a^
X_1_X_5_	33,012.08	1	33,012.08	52.54	0.0004^a^
X_1_X_6_	5123.40	1	5123.40	8.15	0.0290^a^
X_1_X_7_	226.94	1	226.94	0.36	0.5698
X_1_X_8_	1171.43	1	1171.43	1.86	0.221
X_2_X_3_	2369.34	1	2369.34	3.77	0.1002
X_2_X_4_	15,845.51	1	15,845.51	25.22	0.0024^a^
X_2_X_5_	16,444.79	1	16,444.79	26.17	0.002^a^
X_2_X_6_	44,382.89	1	44,382.89	70.64	0.0002^a^
X_2_X_7_	60,608.52	1	60,608.52	96.47	<0.0001^a^
X_2_X_8_	124.01	1	124.01	0.20	0.6724
X_3_X_4_	59,310.66	1	59,310.66	94.40	<0.0001^a^
X_3_X_5_	13,629.19	1	13,629.19	21.69	0.0035^a^
X_3_X_6_	1816.20	1	1816.20	2.89	0.1400
X_3_X_7_	4633.36	1	4633.36	7.37	0.0348^a^
X_3_X_8_	2445.81	1	2445.81	3.89	0.0959
X_4_X_5_	19,617.60	1	19,617.60	31.22	0.0014^a^
X_4_X_6_	2764.93	1	2764.93	4.40	0.0807
X_4_X_7_	1138.43	1	1138.43	1.81	0.2269
X_4_X_8_	4336.96	1	4336.96	6.90	0.0392^a^
X_5_X_6_	6366.15	1	6366.15	10.13	0.0190^a^
X_5_X_7_	981.24	1	981.24	1.56	0.2579
X_5_X_8_	1533.17	1	1533.17	2.44	0.1693
X_6_X_7_	18,457.72	1	18,457.72	29.38	0.0016^a^
X_6_X_8_	5749.72	1	5749.72	9.15	0.0232^a^
X_7_X_8_	3941.71	1	3941.71	6.27	0.0462^a^
X_1_^2^	9150.69	1	9150.69	14.56	0.0088^a^
X_2_^2^	22,642.26	1	22,642.26	36.04	0.0010^a^
X_3_^2^	1708.67	1	1708.67	2.72	0.1502
X_4_^2^	23,864.10	1	23,864.10	37.98	0.0008^a^
X_5_^2^	1138.47	1	1138.47	1.81	0.2269
X_6_^2^	29,138.06	1	29,138.06	46.38	0.0005^a^
X_7_^2^	2082.36	1	2082.36	3.31	0.1185
X_8_^2^	4990.86	1	4990.86	7.94	0.0304^a^
Residual	3769.62	6	628.27		
Lack of Fit	3169.87	2	1584.93	10.57	0.0253 significant
Pure Error	599.75	4	149.94		
Core Total	1.304E+006	50			

The Model *F* value of 47.03 implies the model is significant. There is only a 0.01 % chance that a “Model *F* Value” this large could occur due to noise. Values of “Prob > F” less than 0.0500 indicate model terms are significant

^a^Significant values

1 $$\begin{aligned} {\text{Y}} =\,6 2 4. 20 - 4 9. 7 3 {\text{X}}_{ 1} - 1 6 9. 3 5 {\text{X}}_{ 2} + 5 7. 3 2 {\text{X}}_{ 3} + 20. 6 9 {\text{X}}_{ 6} + 1 8 2. 3 2 {\text{X}}_{ 7} - 4 4. 1 9 {\text{X}}_{ 8} + 5 3.0 8 {\text{X}}_{ 1} {\text{X}}_{ 2} + 1 4 9. 40{\text{X}}_{ 1} {\text{X}}_{ 3} - \hfill 1 70. 30{\text{X}}_{ 1} {\text{X}}_{ 4} + 1 3 9.0 6 {\text{X}}_{ 1} {\text{X}}_{ 5} + 6 8. 4 3 {\text{X}}_{ 1} {\text{X}}_{ 6} - 1 7. 7 5 {\text{X}}_{ 2} {\text{X}}_{ 4} + 8 7. 8 2 {\text{X}}_{ 2} {\text{X}}_{ 5} + 1 1 9. 4 6 {\text{X}}_{ 2} {\text{X}}_{ 6} + 1 5 4. 9 1 {\text{X}}_{ 2} {\text{X}}_{ 7} - 1 9 3. 4 4 {\text{X}}_{ 3} {\text{X}}_{ 4} + 10 2. 4 4 {\text{X}}_{ 3} {\text{X}}_{ 5} - 60. 60{\text{X}}_{ 3} {\text{X}}_{ 7} - 10 5. 9 8 {\text{X}}_{ 4} {\text{X}}_{ 5} - \hfill 6 2. 2 1 {\text{X}}_{ 4} {\text{X}}_{ 8} + 2 8. 1 9 {\text{X}}_{ 5} {\text{X}}_{ 6} - 1 7 9. 6 8 {\text{X}}_{ 6} {\text{X}}_{ 7} - 9 4. 5 3 {\text{X}}_{ 6} {\text{X}}_{ 8} - 6 6. 3 4 {\text{X}}_{ 7} {\text{X}}_{ 8} - 6 3. 3 9 {\text{X}}_{ 1}^{ 2} + 9 9. 7 1 {\text{X}}_{ 2}^{ 2} + 10 2. 3 7 {\text{X}}_{ 4}^{ 2} - 1 1 3. 1 1 {\text{X}}_{ 6}^{ 2} 4 6. 8 1 {\text{X}}_{ 8}^{ 2} \hfill \end{aligned}$$

where Y is the predicted response (Xylanase production); X_1_ is yeast extract, X_2_ is NH_4_NO_3_, X_3_ is peptone, X_4_ is urea nitrogen, X_5_ is CMC, X_6_ is Tween 20, X_7_ is CaCO_3_ and X_8_ is MgSO_4_.

The statistical significance of Eq. (1) was checked by *F* test, and the analysis of variance (ANOVA) for the response surface quadratic model is shown in Table 3. It is evident that the model was highly significant, as suggested by the model *F* value and a low probability value [(*P* model > *F* = 0.0001)]. The ANOVA (*F* test) shows that the second model was well adjusted to the experimental data. The coefficient of variation (CV) indicates the degree of precision with which the treatments were compared. Usually, the higher the value of CV, the lower the reliability of experiment is. Here, a lower value of CV (4.13) indicated a better precision and reliability of the experiments. The precision of a model can be checked by the determination coefficient (*R*^2^) and correlation coefficient (*R*). The determination coefficient (*R*^2^) implies that the sample variation of 97.59 % for xylanase production was attributed to the independent variables, and only about 2.41 % of the total variation cannot be explained by the model. Normally, a regression model having an *R* value higher than 0.9 is considered to have a very high correlation. The closer the value of *R* (correlation coefficient) to 1, the better the correlation between the experimental and predicted values. Here, the value of *R* (0.9971) for Eq. (1) indicates a close agreement between the experimental results and the theoretical values predicted by the model equation. Linear and quadratic terms were both significant at the 1 % level. Therefore, the quadratic model was selected in this optimization study.

The Student *t* distribution and the corresponding *P* value, along with the parameter estimate, are given in Table 3. The *P* values are used as a tool to check the significance of each of the coefficients which, in turn, are necessary to understand the pattern of the mutual interactions between the best variables. The smaller the *P* values, the bigger the significance of the corresponding coefficient. The parameter estimates and the corresponding *P* values suggests that, among the independent variables, X_1_ (yeast extract), X_2_ (NH_4_NO_3_), X_3_ (peptone), X_6_ (Tween 20), X_7_ (CaCO_3_) and X_8_ (MgSO_4_) have a significant effect on xylanase production. The positive coefficients for X_3_, X_4_ and X_7_ indicate a linear effect to increase xylanase production, while negative coefficient for X_1_, X_2_ and X_8_ shows a linear effect to decrease xylanase production. The quadric term of these variables also had a significant effect except X_3_^2^, X_5_^2^ and X_7_^2^. However, no interactions between the X_1_X_7_, X_1_X_8_, X_2_X_3_, X_2_X_8_, X_3_X_6_, X_3_X_8_, X_4_X_6_, X_4_X_7_, X_5_X_7_, X_5_X_8_ variables were found to contribute to the response at a significant level. In this case, X_1_,X_2_, X_3_, X_6_, X_7_, X_8_, X_1_X_2_, X_1_X_3_, X_1_X_4_, X_1_X_5_, X_1_X_6_, X_2_X_4_, X_2_X_5_, X_2_X_6_, X_2_X_7_, X_3_X_4_, X_3_X_5_, X_3_X_7_, X_4_X_5_, X_4_X_8_, X_5_X_6_, X_6_X_7_, X_6_X_8_, X_7_X_8_, X_1_^2^, X_2_^2^, X_4_^2^, X_6_^2^, X_8_^2^ were significant model terms, respectively.

### Comparison of observed and predicted xylanase activity

**Fig. 1:**
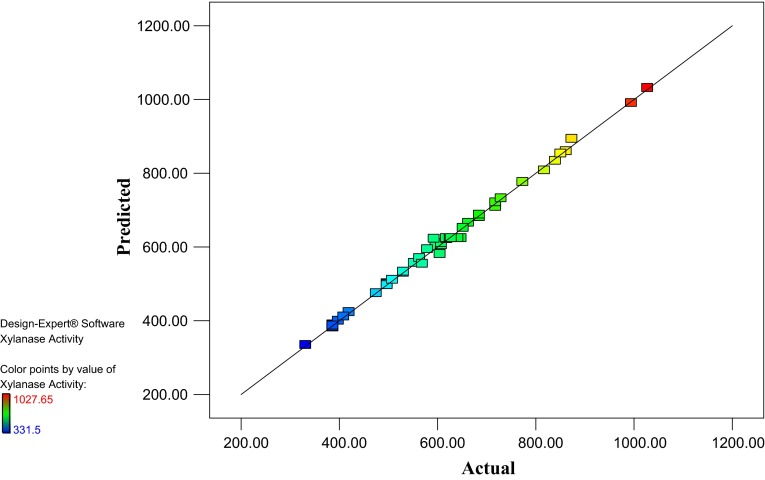
Observed xylanase activity versus the predicted xylanase activity under different fermentation conditions

A regression model can be used to predict future observations on the response Y (xylanase activity) corresponding to particular values of the regressor variables. In predicting new observations and in estimating the mean response at a given point, one must be careful about extrapolating beyond the region containing the original observations. It is possible that a model that fits well in the region of the original data will no longer fit well outside the region. Figure 1 shows observed xylanase activity (the response) versus those from the empirical model Eq. (1). Figure proves the predicted data of the response from the empirical model is in agreement with the observed ones in the range of the operating variables.

### Localization of optimum conditions

The 3D response surface plots described by the regression model were drawn to illustrate the effects of the independent variables on the response variables. The shape of the corresponding contour indicates whether the mutual interactions between the independent variables are significant or not. An elliptical nature of the contour plots indicates that the interactions between the independent variables are significant. From the 3D response surface plots and the corresponding contour plots, the optimal values of the independent variables could be observed, and the interaction between each independent variable’s pair can be easily understood.

**Fig. 2:**
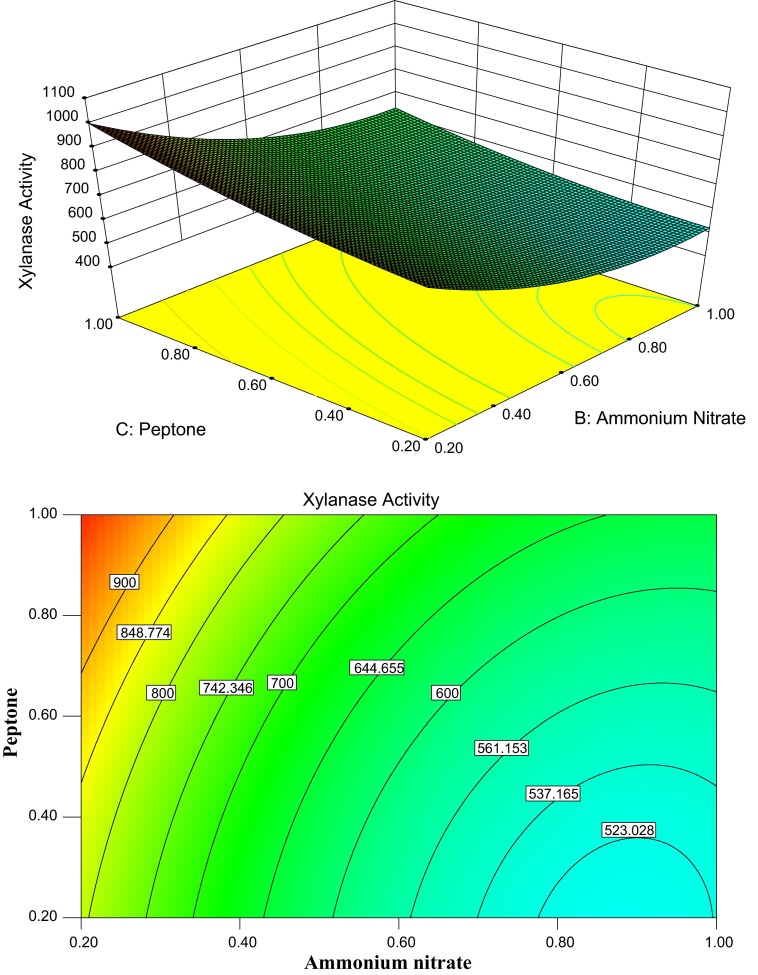
Response surface plot and contour plot of the combined effects of ammonium nitrate and peptone on the xylanase production by *C. cellulans* CKMX1

Figure 2 depicts the 3D plot and its corresponding contour plot, showing the effects of ammonium nitrate concentration and peptone on the xylanase production, while all other six factors were fixed at its middle level. Figure 2 indicates the yield of the xylanase production decreased gradually as the ammonium nitrate concentration increased at a low concentration of peptone. With the increase in the concentration of peptone at its high level, xylanase production significantly increased from 523.02 U/g DBP to 900 U/g DBP at a low initial ammonium nitrate concentration. This suggests that increasing the concentration of peptone within the tested range was beneficial to the accumulation of xylanase production. Our results also show that the increasing of ammonium nitrate concentration beyond 0.20 g (%) decreased the xylanase production.

**Fig. 3:**
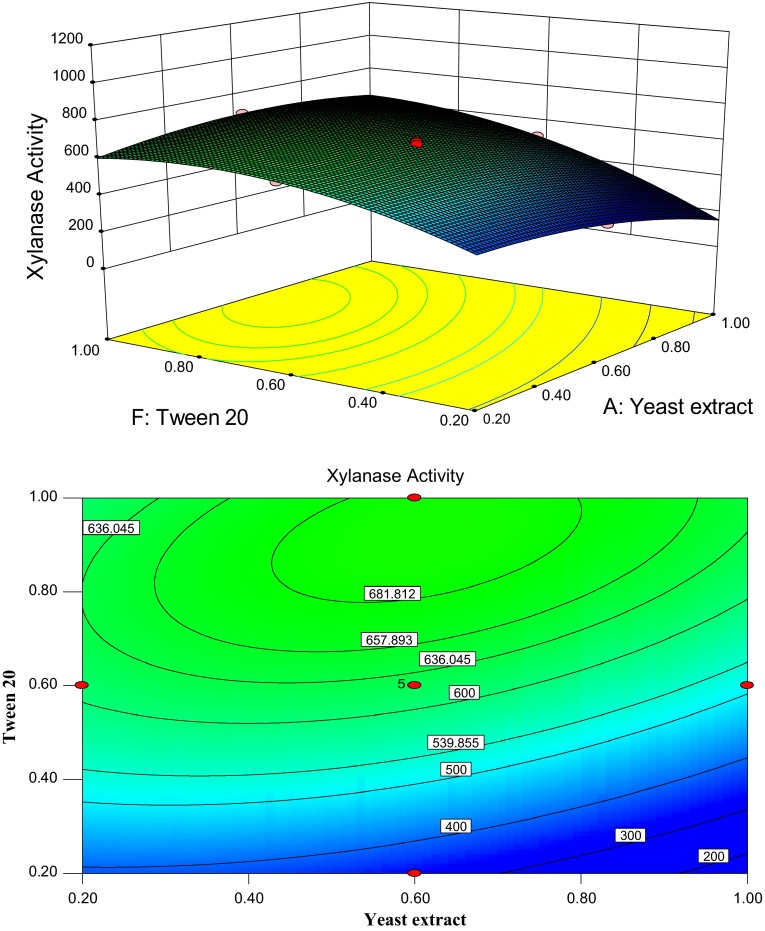
Response surface plot and contour plot of the combined effects of yeast extract and Tween 20 on the xylanase production by *C. cellulans* CKMX1

Figure 3 presents 3D plot and its corresponding contour plot showing the effects of yeast extract and Tween 20 on the xylanase production, while all other six factors were fixed at its middle level. It is evidence that the yield of xylanase production increased simultaneously when both yeast extract and Tween 20 concentration increased, but above 0.60 g (%) concentration of yeast extract xylanase production decreased at a low Tween 20 concentration. This phenomenon was more pronounced when Tween 20 was set at high level and yeast extract was set at middle level, resulting in the change of xylanase production from 400 to 681.81 U/g DBP.

**Fig. 4:**
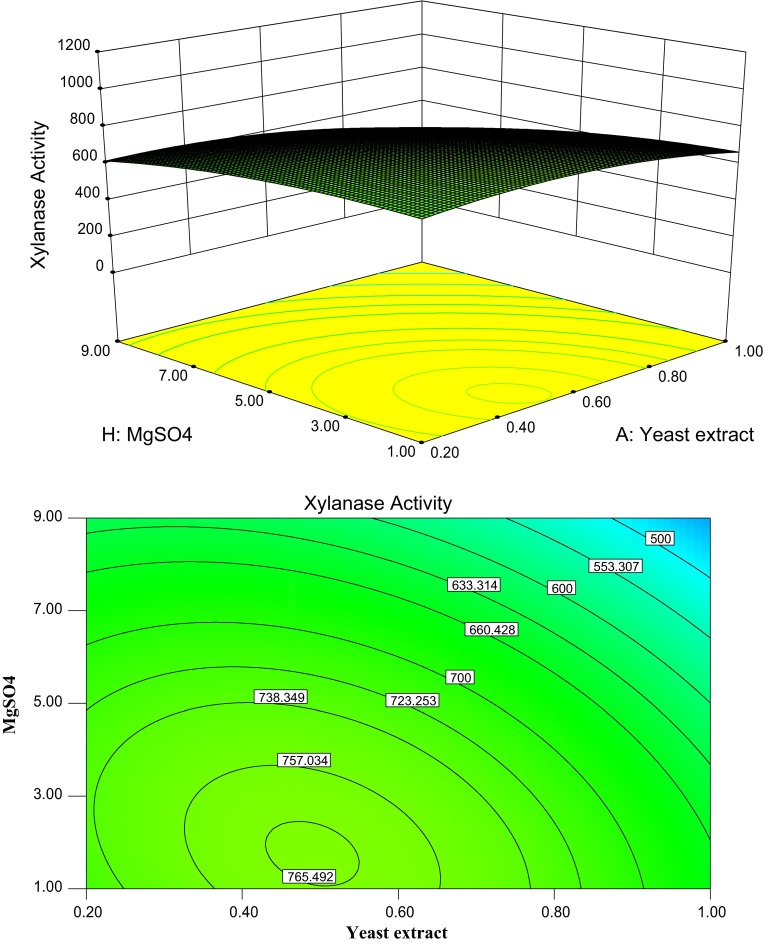
Response surface plot and contour plot of the combined effects of yeast extract and MgSO_4_ on the xylanase production by *C. cellulans* CKMX1

Figure 4 shows the effects of yeast extract and MgSO_4_ on the xylanase production, while all other six factors were fixed at its middle level. When the yeast extract concentration of the cultivation medium was near neutral, increasing the MgSO_4_ concentration to some extent favoured the xylanase production. However, with the increase in the concentration of yeast extract near to neutral, the xylanase production significantly increased from 500 to 765.49 U/g DBP at a low MgSO_4_ concentration. Under this circumstance, the optimum yeast extract concentration and MgSO_4_ concentration were 0.5 g (%) and 3.0 g (%), respectively. However, the xylanase production gradually decreased when yeast extract concentration exceeded optimal conditions 0.5 g (%). This indicated that, under optimal yeast extract and MgSO_4_ concentration, an increase in yeast extract concentration would not further increase the yield of xylanase production. These facts were important in making the whole process economically more feasible.

### Model adequacy checking

**Fig. 5:**
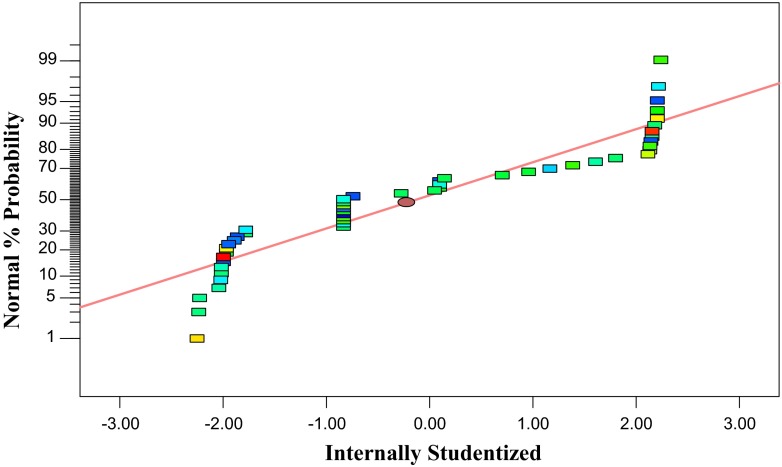
Normal probability of internally studentized residuals

**Fig. 6:**
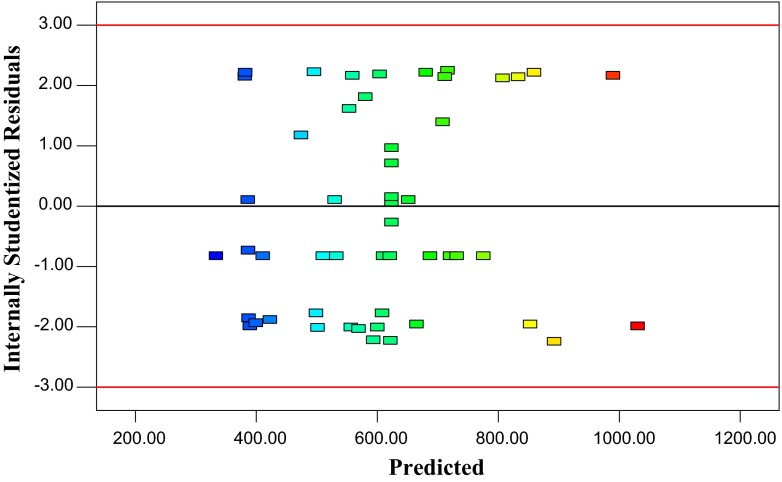
Plot of internally studentized residuals versus predicted response

Usually, it is necessary to check the fitted model to ensure that it provides an adequate approximation to the real system. Unless the model shows an adequate fit, proceeding with the investigation and optimization of the fitted response surface likely give poor or misleading results. The residuals from the least squares fit play an important role in judging model adequacy. By constructing a normal probability plot of the residuals, a check was made for the normality assumption, as given in Fig. 5. The normality assumption was satisfied as the residual plot approximated along a straight line. Figure 6 presents a plot of residuals versus the predicted response. The general impression is that the residuals scatter randomly on the display, suggesting that the variance of the original observation is constant for all values of predicted response (Y). Both of the plots (Figs. 5, 6) are satisfactory, so we conclude that the empirical model is adequate to describe the xylanase activity by response surface.

### Validation of the model

The statistical optimal values of variables were obtained when moving along the major and minor axis of the contour, and the response at low and high level yielded maximum xylanase production. These observations were also verified from canonical analysis of the response surface. The canonical analysis revealed a minimum region for the model. The stationary point presenting a maximum xylanase activity had the following critical values: yeast extract 1.00 g (%), ammonium nitrate 0.22 g (%), peptone 0.91 g (%), urea nitrogen 17.95 mg (%), CMC 4.91 g (%), Tween 20 0.24 mL (%), CaCO_3_ 0.66 g (%) and MgSO_4_ 7.47 g (%). The predicted xylanase activity for these conditions was 1063.50 U/g DBP.

A repeat fermentation of xylanase by *C. cellulans* CKMX1 under optimal conditions was carried out for verification of the optimization. The maximal xylanase level obtained was 1050.24 U/g DBP. This value was found to be 4.07 % less than the predicted value. This discrepancy might be due to the slight variation in experimental conditions. The optimization resulted in 3.1- fold increase of xylanase production, compared with the lowest xylanase production of 331.50 U/g DBP.

## Application of cellulase-free xylanase in pulp biobleaching by *C. cellulans* CKMX1

The kappa number (Tasman and Berzins 1957) of enzyme pre-treated wheat straw pulp was lower than control. At an enzyme dosage of 0.2 and 0.5 %, the enzymatic pre-treatment decreased kappa number by 0.9 points or 7.69 % and 1.4 points or 11.96 %, respectively, as compared with control. In addition to this, enzymatic pre-treatment of 0.2 % of pulp increases the brightness points to 1.1 % ISO, while enzyme dose of 0.5 % of pulp increases the brightness points to 2.2 % ISO. Cellulase-free xylanase from *C. cellulans* CKMX1 under C–EP–D sequence has been shown to bring about a 6.10 % reduction or savings of chlorine in 0.2 % enzyme treated pulp and 12.5 % reduction or savings of chlorine in 0.5 % enzyme treated pulp as compared to control treatment where no enzyme pre-treatment was given, when enzymatically prebleached pulp was charged with 7.4 % of total chlorine. Decrease of 0.5 kappa points or 25 % was observed in treatment where enzyme dose was 0.2 % of pulp, and decrease of 0.8 kappa points or 40 % was found in enzyme treatment of 0.5 % of pulp as compared to control. Paper sheets were prepared using 60 g of pulp on OD basis. Enzyme dose of 0.2 % of pulp increased brightness to 84.71 % ISO points and enzyme dose of 0.5 % of pulp increased the brightness to 85.2 % ISO points as compared to control treatment where brightness was observed to be 83.78 % ISO points. Gain in brightness points was 0.93 % ISO in enzymatic treatment of 0.2 % of pulp and 1.42 % ISO points in enzymatic treatment of 0.5 % of pulp.

## Discussion

Nowadays, there is growing acceptance of the use of statistical experimental designs in biotechnology to optimize culture medium components and conditions (Khucharoenphaisan et al. 2008; Wang et al. 2008; Coman and Bahrim 2011). Many studies have reported satisfactory optimization of xylanase production from microbial sources using a statistical approach (Silva and Roberto 2001; Li et al. 2007a, b; Coman and Bahrim 2011). RSM and CCD were employed to optimize a fermentation medium for the production of xylanase by *C. cellulans* CKMX1 at pH 8.0. The optimization resulted in 3.1-fold increase of xylanase production, compared with the lowest xylanase production of 331.50 U/g DBP (Table 2). Dobrev et al. 2006 also showed that the xylanase activity obtained with the optimized nutrient medium is 33 % higher than the activity, achieved with the basic medium. The application of statistical design for screening and optimization of culture conditions for the production of xylanolytic enzymes allows quick identification of the important factors and the interactions between them (Katapodis et al. 2007; Vasconcelos et al. 2000). The RSM applied to the optimization of xylanase production in this investigation suggested the importance of a variety of factors at different levels. A high degree of similarity was observed between the predicted and experimental values, which reflected the accuracy and applicability of RSM to optimize the process for enzyme production (Techapun et al. 2002; Vasconcelos et al. 2000). The ANOVA (*F* test) shows that the second model was well adjusted to the experimental data. The CV indicates the degree of precision with which the treatments were compared (Wang et al. 2008; Vasconcelos et al. 2000). Usually, the higher the value of CV, the lower the reliability of experiment is. Here, a lower value of CV (4.13) indicated a better precision and reliability of the experiments. The precision of a model can be checked by the determination coefficient (*R*^2^) and correlation coefficient (*R*). The determination coefficient (*R*^2^) implies that the sample variation of 97.59 % for xylanase production was attributed to the independent variables, and only about 2.41 % of the total variation cannot be explained by the model (Table 3). Normally, a regression model having an *R*^2^ value higher than 0.9 is considered to have a very high correlation. The closer the value of *R* (correlation coefficient) to 1, the better the correlation between the experimental and predicted values (Li et al. 2007b). Here, the value of *R* (0.9971) for Eq. (1) indicates a close agreement between the experimental results and the theoretical values predicted by the model equation. Linear and quadratic terms were both significant at the 1 % level. Therefore, the quadratic model was selected in this optimization study. There have been reports on optimization of culture media using statistical approaches for a few bacterial xylanases processes but not for cellulase-free, alkali-stable xylanases in SSF of apple pomace (Li et al. 2007a, b). The statistical optimization approach is efficient and has been applied successfully to SSFs that have overcome the limitations of classical empirical methods (Yu et al. 1997; Ellouze et al. 2008). A response surface method with three-factor-three-level design has been used to optimize the medium components and its pH, for maximum xylanase production by *Bacillus circulans* D1 in submerged fermentation (SmF), which resulted in a maximum concentration of 22.45 U/mL (Bocchini et al. 2002; Senthilkumar et al. 2005). Similarly, xylanase production by *Schizophyllum commune* and *Thermomyces lanuginosus* has been maximized by CCRD method, and the maximum xylanase yields were 5.74 and 2.7 U/mL, respectively, in SmF (Haltrich et al. 1993; Purkarthofer et al. 1993). The results of CCD indicate the significance of yeast extract (X_1_), NH_4_NO_3_ (X_2_), peptone (X_3_), Tween 20 (X_6_), CaCO_3_ (X_7_), and MgSO_4_ (X_8_) on production of xylanase by *C. cellulans* CKMX1. Despite some interactions, maximum interactions of different variables i.e. X_1_,X_2_, X_3_, X_6_, X_7_, X_8_, X_1_X_2_, X_1_X_3_, X_1_X_4_, X_1_X_5_, X_1_X_6_, X_2_X_4_, X_2_X_5_, X_2_X_6_, X_2_X_7_, X_3_X_4_, X_3_X_5_, X_3_X_7_, X_4_X_5_, X_4_X_8_, X_5_X_6_, X_6_X_7_, X_6_X_8_, X_7_X_8_, X_1_^2^, X_2_^2^, X_4_^2^, X_6_^2^, X_8_^2^, respectively, in the present investigation were found to be significant.

Biobleaching processes require xylanases that are active at higher temperature and alkaline pH. The crude xylanase from *C. cellulans* CKMX1 showed high thermostability (up to 60 °C) over a broad pH range (5–10).The study of the physical and chemical properties of a pulp prebleached with enzyme charge of 0.2 and 0.5 % pulp for 2 h before chemical bleaching treatment revealed an increase in the brightness points by 1.1 and 2.2 % ISO and an increase in residual chlorine by 6.10 and 12.5 %. In a previous study using 10 IU/g commercial xylanase P, the control level of brightness was retained at 23.3, 30 and 16.7 % ClO_2_ reduction for bagasse, soda-aq, and Kraft pulps, respectively (Madlala et al. 2001). In another study, the use of commercial xylanases Novozyme 473 and VAI-Xylanase increased brightness of Kraft pulp by 2.5 points at 31 % ClO_2_ reduction (Bajpai et al. 1994; Singh et al. 2013). The effectiveness of xylanase treatment before chemical bleaching application may be due to cleavage of either the linkage of residual lignin to hemicellulose, leading to increased accessibility of the pulp to bleaching chemicals and enhanced extraction of lignin, or target substrate modification during subsequent bleaching stages (Ninawe and Kuhad 2006; Azeri et al. 2010).

## Conclusion

Statistical optimization of cultivation conditions using the central composite appeared to be a valuable tool for the production of xylanase by *C. cellulans* CKMX1. The predicted and actual xylanase activity under optimal conditions in stationary flasks experiments were 1041.93 U/g DBP and 1027.65 U/g DBP, respectively. A scale-up of the fermentation process was carried out in a aluminium trays to reconfirm the maximum xylanase activity of 1150.37 U/g DBP after 72 h cultivation under optimized conditions. Cellulase-free xylanase from *C. cellulans* CKMX1 under C–E_P_–D sequence has been shown to bring about a 12.5 % reduction of chlorine, decrease of 0.8 kappa points (40 %) and gain in brightness was 1.42 % ISO points in 0.5 % enzyme treated pulp as compared to control where no enzyme pre-treatment was given, when enzymatically prebleached pulp was charged with 7.4 % of total chlorine. From the present studies, it is clear that *C. cellulans* CKMX1 xylanase is having the characteristic suited for an industrial enzyme.
